# Medical News and Miscellany

**Published:** 1887-03

**Authors:** 


### MEDICAL NEWS AND MISCELLANY.

Cincho Quinine produces much less constitutional disturbance; and in the same doses, the therapeutical effects are fully equal to the sulphate.

### A GENERAL INVITATION.

The JOURNAL desires to call attention to the spirited address of President Nott, of the Texas State Medical Association, copies of which were mailed from this office to over one thousand physicians in the State—to many a number of copies for distribution,—and to remind readers that this is not to be a *convention of members only*, but a rallying of the profession around the standard raised by the Association—for the advancement of medical science in this great

State,—the nucleus around which it is hoped to organize the entire regular profession. Hence, we say—do not stay away because you are not a member,—come and become a member,—and join in the good work. An attractive programme has been arranged for your entertainment, and the recreation obtained by a week's rest from the routine of practice will infuse new life, new energy, and new ambition into each one, and your profession will seem dearer to you than ever before.

---

"TEXAS SURGERY."

Doubtless the near approach of the annual meeting of the Texas State Medical Association has deterred many members from paying their arrears, as it is a very general thing to pay at the meeting. But, in consequence of this failure, the Secretary has not been able to distribute this very valuable document. An effort is now being made to get them speedily into the hands of members, and we will suggest that assistance in the way of dues will, at any time, be acceptable. The postage on the lot will amount to a considerable sum. We are much gratified at the reception given the few copies which have been issued. The committee (Dr. Cupples, chairman) have received letters of warm commendation from the most distinguished medical men of Europe and America. Amongst the lights who have complimented the work, we may be pardoned for mentioning, Bowditch, Gregory, Lawson Tait, S. W. Gross, Senn, Battey, Richardson, Barker, Bodenhamer, Pepper, Ashurst, Packard, Sinclair, T. G. Thomas, W. L. Lee, aud others.

This great compliment—the endorsement of the whole profession of America and part of Europe, should stir the pride, and stimulate the zeal of our surgeons, and induce them to contribute more freely and fully, their cases. The work will be continued; and though the medical history of the past may never be written; a mistaken notion of propriety may prevent many of the now pioneers of Texas medicine from giving their biography to the world—this, we repeat, will be a monument to their worth and ability,—the one stone left to tell that they once lived and labored.

Under the head of "Texas Surgery," we may mention that every day creditable operations are being performed by our surgeons all over the State—operations which should be recorded. Dr. Cupples recently performed laparotomy for g. s. wound of abdomen, with resection of intestine,—the first case, we venture to assert, south of the Potomac. The patient, however, died of the shock and antecedent hemorrhage. We note a case of imperforate hymen and retained menses lately reported by Dr. Ghent, in the *Texas Courier Record.* A similar case was operated on by an Austin physician last week. We are promised a report of Dr. Cupples' case, and hope to be able to give the details in our next issue.

---

THE TEXAS DENTAL BILL.

This bill, drawn and presented by the Texas Dental Association, is now before the Legislature, and has passed to engrossment. It is thought it will become a law. It provides, first, that no person shall practice dentistry in this State without a license from an examining board, to be appointed under this act. (Dentists who have been in regular practice for the three years next preceding the passage of the act, and graduates of incorporated dental colleges—are exempt from examination, but the latter must exhibit their diploma and procure license; the former must furnish satisfactory evidence that they have been in regular practice for the time required, and thus obtain a license without fee.) Second. The President, Secretary and Executive Committee of the Texas Dental Association shall constitute the Board. Third. The Board shall appoint a district examiner in each congressional district, who shall be a resident of that district, and a member of the Texas Dental Association. Fourth. The provisions of the act do not apply to physicians and surgeons practicing as such, nor is it intended to prevent other persons from merely extracting teeth. The penalty for violation is not less than $100 nor more than $200. All fines for violation of act to go to the common school fund of the county where collected.

---

TEXAS CARRIES OFF THE HONORS AS USUAL.

At the 50th annual commencement of the Medical Department of the University of Louisville, Kentucky, in a graduating class of eighty-seven, there were seventeen Texans. On the roll of honor consisting of ten best, there were three Texans, to-wit: 1st honor, Jno. L. Dodge; 5th honor, E. P. Montgomery; and 6th honor, S. E. Milliken.

Dr. Jno. L. Dodge, the first honor man, won the first prize—the

Yandell gold medal for highest class standing· also, Prof. Ouchterlony's prize, a gold medal for the best report of Professor O's clinical lectures; also, Prof. Anderson's prize—a case of obstetrical instruments, for the best examination in obstetrics and diseases of women and children; also Prof. Bailey's prize—a gold medal, for best examination in materia medica, therapeutics and public hygiene—honors enough for one day surely.

In the undergraduates contest W. R. Spalding of Austin, Texas, won 1st prize, a copy of Gross' Surgery, and also won Prof. Palmer's prize, a gold medal for best examination in physiology; also one of the two free scholarships for next session—offered by the faculty for the two first course students who should pass the best examination in all branches; the other scholarship being awarded to S. Z. Bryan, also a Texan.

We would be glad to be favored with the address of these young gentlemen, who have so distinguished themselves and their State. Mr. Spalding is a student here, in the office of Dr. McLaughlin, and was formerly we believe with Dr. Wooten.

---

We are Pleased to announce large accessions to our subscription list lately, coming principally, from the East, where organization is on a boom. Several new county societies have sent us quite a list of new subscribers, and we want to tender our acknowledgement particularly to Dr. J. E. Mayfield of Nacogdoches, Secretary East Texas Medical Association, and to Dr. W. Y. Jameson of Rusk, for favors in this line; also Dr. J. T. McCarty, Canton, Secretary, Kaufman County Medical Society.

---

### SUBSTITUTION IN PRESCRIPTIONS.

The *St. Louis Medical and Surgical Journal* has a well-timed editorial on this nefarious practice on the part of some druggists, in which the writer points out that by the practice not only are manufacturers of proprietary and official preparations swindled out of their just dues, but the reputation of the physician who prescribes, and the safety of the patient prescribed for, are both jeopardized. A moments reflection on the part of any physician will convince him of the correctness of this statement. One article, which has been tried, and is known to be good, is ordered, and "something just as good" is substituted by the druggist. The prescription fails of its object, time is lost which cannot be recalled, and the Doctor loses faith in the article he ordered—but did not get. It is gratifying to see the medical press speaking out in condemnation of this unwarranted assumption of responsibility and authority on the part of druggists, and a physician should cease to send his prescriptions to any man who would *dare* do it.

---

TRANSACTIONS TEXAS STATE MEDICAL ASSOCIATION, '86.

**(A few brief Extracts from Reviews of the Work.)**

Of this volume, *The Mississippi Valley Medical Monthly* says:

"It is a surprise, from the fact that in appearance, it far exceeds in general makeup, the average 'Transactions' from any of our State Societies. It contains nearly 700 pages of well written and well edited reading matter," etc.

The *Louisville Herald* says:

"This report of the Transactions of the Texas Association is a most creditable production, and shows that the physicians of that great State are fully conscious of their necessities and welfare, and are in every respect the peers of their professional brethren elsewhere. Every Texan that we have seen brags more than a Kentuckian, if possible, about his State—its natural resources, its immensity, and its future prospects. He can now brag about its doctors."

The *American Lancet* says:

"The size of the work—nearly 700 pages—shows the time (four days) has been spent in actual work. The paper, presswork and binding are models of good taste. * * * Altogether, the profession of Texas must be congratulated upon this showing of her State Medical Association. For its opportunities, no State in the Union has done better; few nearly so well."

*Progress*, Louisville, Ky., says: (From a six column review.)

"This volume is the largest, and in every way, the most creditable work of its kind in this country. No other State can offer anything for favorable comparison with it."

